# Production, purification and characterization of a thermotolerant alkaline serine protease from a novel species *Bacilluscaseinilyticus*

**DOI:** 10.1007/s13205-016-0377-y

**Published:** 2016-02-13

**Authors:** Thirumala Mothe, Vishnuvardhan Reddy Sultanpuram

**Affiliations:** Microbial Ecology Lab, Department of Biochemistry, Mahatma Gandhi University, Anneparthy, Yellareddygudem (PO), Nalgonda, 508254 Telangana India

**Keywords:** Protease, Assay, Application, Laundry detergent industry

## Abstract

Alkaline proteases are important enzymes in many industrial applications, especially as additives in laundry detergent industry. Though there are a number of *Bacillus* species which are reported to be producing proteases, the efficiency of a protease produced by a novel strain has to be studied in comparison to the others. Hence, in this study, an alkaline serine protease produced by a novel species *Bacillus* *caseinilyticus* was purified and characterized for its possible usage in detergent industry. Ammonium sulphate, dialysis and DEAE column chromatographic methods were used for purification of the isolated alkaline protease. The molecular weight of the protease was determined by SDS-PAGE and it was found to be 66 kDa. Peptide mass fingerprinting (PMF) was carried out using MALDI-TOF-TOF mass spectrometry and the peptides were found to be similar to that of subtilisin protease. Specific activity of purified protein was found to be 89.2 U/mg. Optimum pH and temperature for enzyme activity were at pH 8 and 60 °C, respectively, showing stability with 10 mM CaCl_2_. Phenyl methyl sulphonyl fluoride (PMSF) at both 5 and 10 mM concentrations completely inhibited the enzyme activity suggesting its serine nature. EDTA, metal ions Mg^2+^ and Ca^2+^ increased the enzyme activity. The one factor at a time optimisation of the protease production was carried to identify the important factors that affect its production. After optimisation, the protease was produced at lab scale, purified and characterised. This alkali, thermotolerant serine protease was found to be significantly stable in the presence of various surfactants and H_2_O_2._ Also, it was successfully able to remove blood stain when used as an additive along with commercial detergent suggesting its potential application in the laundry detergent industry.

## Introduction

Nearly two-third of commercial proteases are produced by fungal species, yeasts and bacteria (Wang et al. 2013). Proteases have diverse applications in various industries, such as, food, pharmaceuticals, silk and diagnostics with predominant use in detergent and leather industries (Banik and Prakash 2004; Gupta et al. 2002). The demand for industrial enzymes, particularly of microbial origin, is ever increasing owing to their applications in wide variety of processes. Proteases represent one of the three largest groups of industrial enzymes and account for about 60 % of the total worldwide sale of the enzymes (Rajkumar et al. 2011).

Proteases are broadly classified as endo or exoenzymes on the basis of their site of action on protein substrates. They are further categorized as serine proteases, aspartic proteases, cysteine proteases or metalloproteases depending on their catalytic mechanism (Geethanjali and Subash 2011). Alkaline serine proteases of microbial origin possess considerable industrial potential due to their biochemical diversity and wide applications in tannery, food industries, medicinal formulations, detergents and processes like waste treatment, silver recovery and resolution of amino acid mixtures (Agarwal et al. 2004).

*Bacillus* species are specific producers of extracellular protease. Several alkaline proteases have been purified and characterized from many *Bacillus* strains (Rao et al. 1998). Subtilisin Carlsberg produced by *Bacillus liceniformis* (Jacobs et al. 1985) and Subtilisin Novo produced by *Bacillus amyloliquefaciens* (Wells et al. 1983) have been the enzymes of choice for detergent industries. These enzymes exhibit maximum activity at alkaline pH values ranging from 8 to 10 (Horikoshi 1999). Generally the alkaline proteases for detergent applications should be active at temperature higher than 40–50 °C and pH in the range of 9–12 (Sellami-Kamoun et al. 2008; Hadder et al. 2009).

The purification process also increases the specific activities of enzymes, making them more specific for industrial applications. In the present study, we report purification and characterization of a thermotolerant alkaline serine protease produced by a novel species, *Bacillus caseinilyticus*.

## Materials and methods

### Materials

Bovine Serum Albumin (BSA), reagents for protein estimation and SDS-PAGE, DEAE-Cellulose were purchased from Hi-Media, Mumbai, India. Silica gel TLC plates (0.25 mm), trichloroacetic acid (TCA), casein and other analytical grade chemicals were purchased from Merck, Mumbai, India.

### Microorganism

A novel *Bacillu*s strain SP^T^ was isolated from the alkaline Lonar Lake, located at Buldhana, Maharashtra, India. The detailed taxonomic characterisation and identification of this strain proposed as a novel species, *Bacillus caseinilyticus* has been described elsewhere (Vishnuvardhan Reddy et al. 2015).

### Optimization of culture conditions and media for protease production: one-factor-at-a time

Production of protease by *Bacillus caseinilyticus* was carried out in basal medium with the following composition (g l^−1^): yeast extract, 0.01; KH_2_PO_4_, 0.5; MgSO_4_·7H_2_O, 0.2; (NH_4_)_2_HPO_4_, 1.0; NaCl, 60 with different combinations of carbon and nitrogen substrates (1 % w/v) and 1 % inoculum size at 37 °C for 48 h in a rotary shaker (180 rpm). The initial pH of the medium was adjusted to 8.0. Parametric optimization was performed with respect to substrates (both carbon and nitrogen), metal ions, pH, temperature, fermentation period and their individual effects were monitored. The cell-free supernatant was recovered by centrifugation at 10,000 rpm for 10 min at 4 °C and used for determining extracellular protease activity.

For screening significant variables affecting protease production by *Bacillus caseinilyticus*, various carbon (1 % w/v; glucose, sucrose, lactose, fructose and maltose), nitrogen (1 % w/v; ammonium chloride, malt extract, yeast extract, peptone and skim milk) substrates, metal chlorides (CaCl_2_, MgCl_2_, ZnCl_2_, FeCl_2_ and CuCl_2_) at 5 mM concentration, incubation time (24, 48, 74, 98 and 120 h), temperature (20, 30, 37, 40, 50, 60 and 70 °C) and pH (6, 7, 8, 9, 10, 10.5 and 12) were tested by one-factor-at-a time strategy. Controls were prepared simultaneously to compare the data of tests without adding any carbon, nitrogen sources or metal chlorides.

### Enzyme assay

Alkaline protease activity was determined with the method of Lin et al. (1969) using *N*,*N*-dimethylated casein (DMC) as a substrate. The reaction mixture containing 2 ml of 1 % (w/v) DMC in 100 mM borate-hydrochloride buffer (pH 8), 0.25 ml of 1 % 2,4,6-trinitrobenzene sulfonic acid (as a colour indicator) and 1 ml of cell free extract with enzyme was incubated at 60 °C for 25 min with constant shaking. After incubation the reaction was stopped by adding 2.5 ml cold water for 15 min. The precipitate was then removed by centrifugation at 10,000×*g* for 15 min at 4 °C and absorbance was measured at 450 nm against blank. The amount of the enzyme which catalysed the cleavage of 1 μmol of peptide bond from DMC per minute under the experimental conditions used was defined as one proteolytic unit (IU).

### Protein estimation

Total protein content was determined by Lowry et al. (1951) using bovine serum albumin as reference.

### Protease purification

#### Ammonium sulphate precipitation

Ammonium sulphate precipitation was the method by which cell free supernatant was precipitated using different saturation levels of ammonium sulphate (40, 50, 60, 70 and 80 %). After each addition, the enzyme solution was stirred for 1 h at 4 °C. The precipitated protein was separated by centrifugation at 12,000×*g* for 20 min at 4 °C and resuspended in small volume of 0.05 M Tris–HCl buffer, pH 8.0 to get the concentrated enzyme suspension. The precipitate was harvested by centrifugation at 10,000×*g* for 15 min, than dissolved in 20 mM Tris–HCl buffer (pH 8.8) and dialysed against the same buffer overnight at 4 °C.

#### Purification of protease by DEAE cellulose column chromatography

Further purification was carried out using DEAE cellulose column chromatography, for which 100 ml dialysed enzyme was applied to DEAE-cellulose column (2.4 × 45 cm) pre-equilibrated with 0.05 M Tris–HCl buffer (pH 8). The enzyme was eluted with the same buffer at a flow rate of 20 ml/h.

#### SDS-polyacrylamide gel electrophoresis and peptide mass spectrometry

The molecular weight of the alkaline protease was determined by running the SDS-PAGE against standard molecular weight protein marker including phosphorylase b (97.4 kDa), bovine serum albumin (66 kDa), egg ovalbumin (45 kDa), and carbonic anhydrase (29 kDa) and analysing after band formation (Laemmli 1970). The gels were stained with Coomassie Brilliant Blue R-250 in methanol–acetic acid–water (5:1:5, v/v), and decolorized with 7 % acetic acid. Further, the molecular mass of the peptides of the purified protein were analyzed by the method of matrix assisted laser desorption ionization-time of flight-time of flight (MALDI-TOF-TOF) mass spectrometry. The instrumentation used was the Applied Biosystems (AB) Model 4800 MALDI-TOF/TOF mass spectrometer. Reflectron MS analysis sums 1250 laser shots to generate the peptide fingerprint map (PFM) and the spectra were internally calibrated using the bradykinin as an internal standard. Masses were chosen by the AB 4000 Series Explorer software (version 3.0) for MS/MS acquisition.

#### Effect of pH and temperature on enzyme activity and stability

To determine the effect of pH and temperature on protease activity, the reaction mixtures [diluted enzyme (10 µl of purified enzyme + 190 µl buffer) + 1 % (w/v) DMC as a substrate] were incubated at different pH values (6–12) and temperatures (30–100 °C). The different buffers used were: (0.05 M) phosphate (pH 6–7), Tris–HCl (pH 8–9) and glycine–NaOH (pH 10–12). To check the pH stability 10 µl of enzyme and 190 µl of the above said buffer solutions were incubated. The residual activities were then measured according to the standard assay method. To determine the stability, the enzyme was preincubated for 24 h at the above mentioned reaction conditions with 5 mM and 10 mM CaCl_2_.

#### Concentration determination by active site titration

The active site titration was performed using *p*-nitrophenyl *p*′-guanidinobenzoate (PNPGB). A 10 mM PNPGB stock solution in dimethyl formamide (DMF) was prepared. To 990 µL purified enzyme solution, 10 µL 10 mM PNPGB was added, mixed quickly and thoroughly and OD was measured immediately at 410 nm (Chase and Shaw 1969).

#### Effect of inhibitors and metal ions on protease activity

Effect of various inhibitors, such as, PMSF (phenyl methyl sulphonyl fluoride) at 5 mM and 10 mM concentrations, ethylene diamine tetra acetic acid (EDTA) and metal ions (Ca^2+^, Mg^2+^, Zn^2+^, Co^2+^, Fe^2+^, Na^2+^, Cu^2+^ and Ni^2+^) at 5 mM concentrations on protease activity were studied. The purified enzyme was preincubated with the above mentioned inhibitors and metal ions (10 µl enzyme + 190 µl 500 mM Tris–HCl buffer and 1 % (w/v) DMC as substrate) for 1 h at 60 °C and then assayed for residual activity. Controls were also run without inhibitors and metal ions along with tests.

#### Effect of different substrates on enzyme activity

The effect of different substrates on the alkaline protease was checked using various substrates like, casein, gelatin, bovine serum albumin (BSA) and egg albumin. The reaction mixture containing 200 µl of enzyme and 200 µl of substrate (1 mg/ml) was incubated at 60 °C for 20 min and the activity was estimated using standard assay.

#### Effect of polar and non-polar solvents on protease activity

The effect of solvents on enzyme activity was studied by incubating the enzyme with (10 µl enzyme + 190 µl 500 mM Tris–HCl buffer and 1 % (w/v) DMC as substrate) the polar and non-polar solvents (10 % v/v), such as, acetone, benzene, chloroform, hexane and toluene for 24 h at 60 °C and than assayed for residual activity of the enzyme. Controls were run along with the tests.

#### Effect of surfactants and different oxidizing agents on stability of protease

Effect of Tween 20 at 0.5 %, 1 %, SDS at 0.5 %, 1 % and H_2_O_2_ at 0.5 % and 1 % were studied on protease activity by incubating the mixtures (10 µl enzyme + 190 µl 500 mM Tris–HCl buffer and 1 % (w/v) DMC as substrate) for 1 h at 60 °C. Later the residual activities were measured; controls were used in studies without surfactants and oxidizing agent.

### Statistical analysis

All the experiments were performed in triplicate each time. The mean ± standard deviation (SD) and Spearman’s rank correlation coefficient was employed on the data for protease production, protease activity and stability at different temperatures and pH concentrations were tested for their significance using Microsoft Excel.

### Application of purified protease on blood stain removal

Application of the purified protease as a detergent additive in blood stain removal was studied on white cotton cloth pieces (10 cm × 10 cm) stained with blood and oven dried at 95–100 °C for 5 min. The stained cloth pieces were taken in separate trays. The following sets were prepared and studied:

- Tray with distilled water (100 ml) + blood stained cloth + 1 ml of commercial detergent (Surf excel-5 mg/ml) + 2 ml of purified enzyme.
- Tray with distilled water (100 ml) + blood stained cloth + 1 ml of commercial detergent (Surf excel-5 mg/ml).
- Untreated cloth piece stained with blood was considered as control.
- Tray with distilled water (100 ml) + blood stained cloth + 2 ml of purified enzyme.
- Tray with distilled water (100 ml) + blood stained cloth.

The trays were incubated at 50 °C for 30 min. The cloth pieces were taken out from each set at regular intervals of 5 min, rinsed with water, dried and visually examined.

## Results and discussion

### Optimization of culture conditions and media for protease production: one-factor-at-a time

The organism was able to produce protease at a pH range of 7.0–10.0 (optimum production at pH 9.0) and at a temperature range of 30–60 °C (optimum production at 37 °C). Among the carbon sources tested, fructose showed more production ability, followed by sucrose. Among different organic nitrogen sources, skim milk gave maximum protease yield followed by malt extract, peptone and yeast extract. Ammonium chloride as inorganic nitrogen source was found to inhibit the production. MgCl_2_ and CaCl_2_ induced protease production, whereas, other metal salts like ZnCl_2_, FeCl_2_ and CuCl_2_ had minimal effect. Increase in incubation time enhanced protease production till 48 h. But, gradual decrease in enzyme production was observed with prolongation of fermentation time (Table 1).

**Table 1:** Effect of fermentation period, pH, temperature, carbon sources, nitrogen sources and metal chlorides on alkaline serine protease production by *Bacillus* *caseinilyticus*

Fermentation period (h)	Protein concentration (mg/ml)
26	39.30 ± 2.5
48	42.67 ± 3.5
74	34.65 ± 4.0
98	28.76 ± 2.8
120	24.35 ± 2.1
pH	
6	0
7	29.30 ± 3.5
8	33.67 ± 2.5
9	49.76 ± 1.5
10	28.16 ± 2.6
10.5	0
12	0
Temperature (°C)	
20	0
30	39.10 ± 2.5
37	49.99 ± 3.5
40	39.76 ± 1.5
50	28.16 ± 2.9
60	20.22 ± 1.8
70	0
Carbon sources	
Glucose	33.55 ± 1.9
Sucrose	42.66 ± 1.8
Lactose	39.16 ± 1.8
Fructose	48.16 ± 1.9
Maltose	21.92 ± 1.7
Nitrogen sources	
Ammonium chloride	0
Malt extract	43.99 ± 1.9
Yeast extract	31.19 ± 1.2
Peptone	39.19 ± 1.0
Skim milk	47.92 ± 2.1
Metal chlorides	
CaCl_2_	42.35 ± 2.1
MgCl_2_	42.55 ± 3.9
ZnCl_2_	39.77 ± 2.8
FeCl_2_	38.68 ± 2.9
CuCl_2_	31.29 ± 1.8
Control (only basal medium)	12.45 ± 3.7

All the results were presented as mean ± SD

The optimized parameters were used for the production of the enzyme in the medium with fructose as the carbon source and skim milk powder as the nitrogen source, with MgCl_2_ and CaCl_2_ as additional additives. The final pH of the medium was adjusted to 9.0 and the organism was grown at 37 °C for 48 h.

### Purification and mass peptide spectroscopy of alkaline protease

**Fig. 1:**
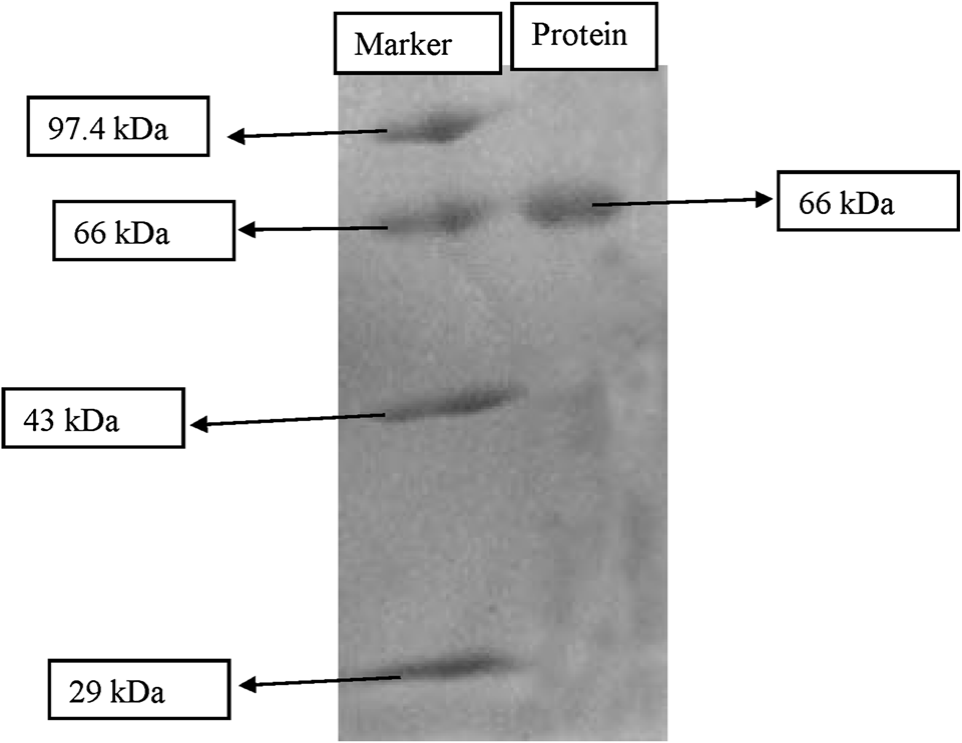
SDS PAGE of the purified alkaline serine protease of *Bacillus* *caseinilyticus*

After production of enzyme, it was precipitated optimally using ammonium sulphate at 60 % saturation level. The ammonium sulphate saturation increased the protein purification 1.65 fold with 60 % recovery; later on enzyme was purified by dialysis method. The dialysed sample was further purified by DEAE cellulose column chromatography. The purification of protein using the DEAE cellulose column chromatography increased the purity of the protein by 20.74-fold compared to ammonium sulphate precipitation and dialysis (Table 2). Specific activity of purified protein was 89.2 U/mg. Enzyme purity was confirmed by SDS-PAGE with the help of standard protein marker run against the test sample. The molecular weight of the protein was found to be 66 kDa (Fig. 1). A 34 kDa serine protease from *B. pumilus* CBS, and a 35 kDa manganese-dependent alkaline serine protease from *B. pumilus* TMS55 have been previously reported (Jaouadi et al. 2008; Ibrahim et al. 2011). Moreover, a 38 kDa organic solvent and detergent stable protease was also reported from *Bacillus* sp. RKY3 (Reddy et al. 2008). The molecular weight of the isolated enzyme was in the range of some reported *Bacillus* proteases.

**Table 2:** Summary of purification of alkaline serine protease produced by *Bacillus* *caseinilyticus*

Purification method	Total activity (U)	Total protein (mg/ml)	Specific activity (U/mg)	Purification fold	Yield (%)
Crude enzyme	5.93	1.38	4.3	1.00	100
Ammomium sulphate precipitation	8.52	1.20	7.1	1.65	48
Dialysis	9.60	1.00	9.6	2.23	35
DEAE cellulose column chromatography	11.59	0.13	89.2	20.74	18

**Fig. 2:**
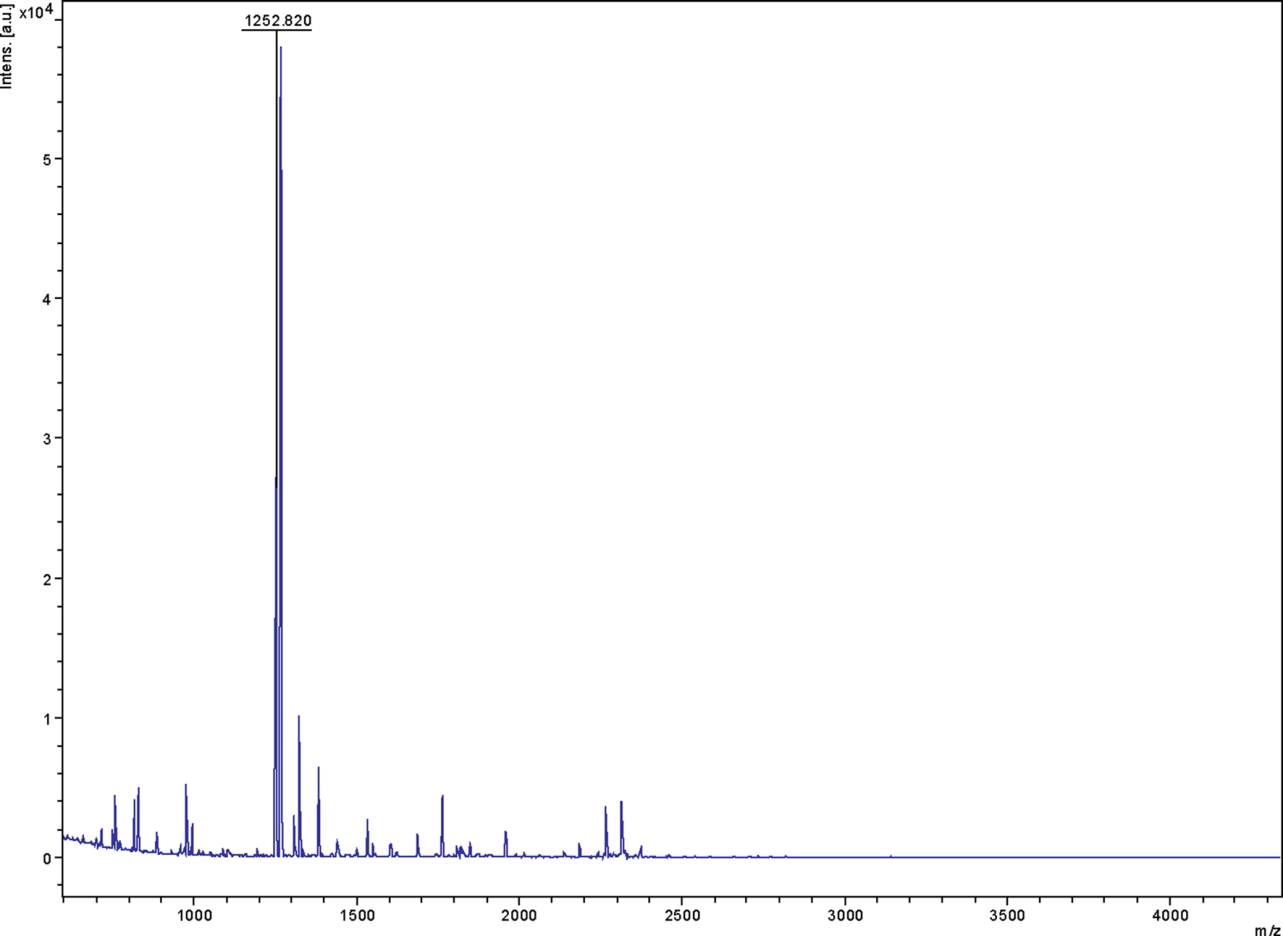
PFM spectra of alkaline serine protease from *Bacillus* *caseinilyticus*

The peptide mass fingerprinting (PMF) of the protein after digestion with trypsin yielded eight prominent *m*/*z* peaks (Fig. 2). The *m*/*z* values corresponding at 1958.2, 1324.6 and 1308.9 were identified as peptides with sequences LEAAPVMFPERPAYPDR, GVAPDAEIYAYR and LIGETIADFSSR, respectively. These peptides were similar to peptidase S8 and S53 subtilisin from *Bacillus cellulosilyticus*.

### Effect of pH and temperature on alkaline protease activity and stability

**Fig. 3:**
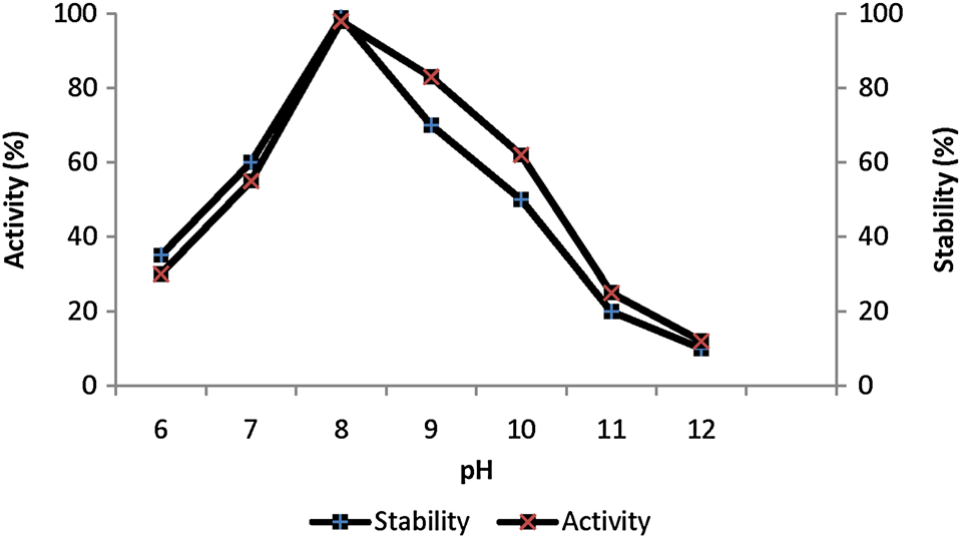
Effect of pH on activity and stability of alkaline serine protease of *Bacillus* *caseinilyticus*

The enzyme exhibited activity in the wide range of pH from 6 to 10 with the maximum activity at pH 8 (*P* ≤ 0.1), which supports that the enzyme with alkaline nature. Some of the *Bacillus* derived proteases viz. from *B. subtilis* NCIM 2713 reported by Mane and Bapat, had an optimum activity at pH 8 and was stable at pH 6.5–9 (Mane and Bapat 2001). The protease from *B. subtilis* VSG-4 reported by Giri et al. (2011) had the maximum activity at pH 9 with a sharp decline in the activity in pH values lower than pH 9. An extracellular hrtA-like serine protease by *B. subtilis* DR8806 exhibited high activity in the range of pH from 5 to 10 with the maximum activity at pH 8 (Farhadian et al. 2015). Similarly, a halophilic bacterium isolated from sea water catalyzed reactions in the pH range 8–11 and performed optimally at pH 10 (Raval et al. 2014). Enzyme isolated in this study was more stable at pH 8 in presence of 10 mM CaCl_2_ (Fig. 3).

Effect of different temperatures showed that the enzyme was active at these varied temperatures, however, optimum temperature was found to be 60 °C (*P* ≤ 0.1) supporting thermotolerant nature of the enzyme. Maximum proteolytic activity of *Bacillus* strains HR-08 and KR-8102 isolated from soil of western and northern parts of Iran have been recorded at 65 and 50 °C, respectively (Moradian et al. 2006). A serine protease by *B. subtilis* DR8806 showed highest activity at 45 °C and withstood at temperature up to 70 °C (Farhadian et al. 2015). Cha et al. (2005) reported that the protease from *Bacillus* sp. SS103 was active at 37 °C and the alkaline protease from *B.subtilis* VSG-4 that previously reported by Giri et al. (2011) was active over a range of temperature from 40 to 60 °C with an optimum temperature at 50 °C. In addition, Huang et al. purified an alkaline protease that had maximum activity at 55 °C (Huang et al. 2003).

**Fig. 4:**
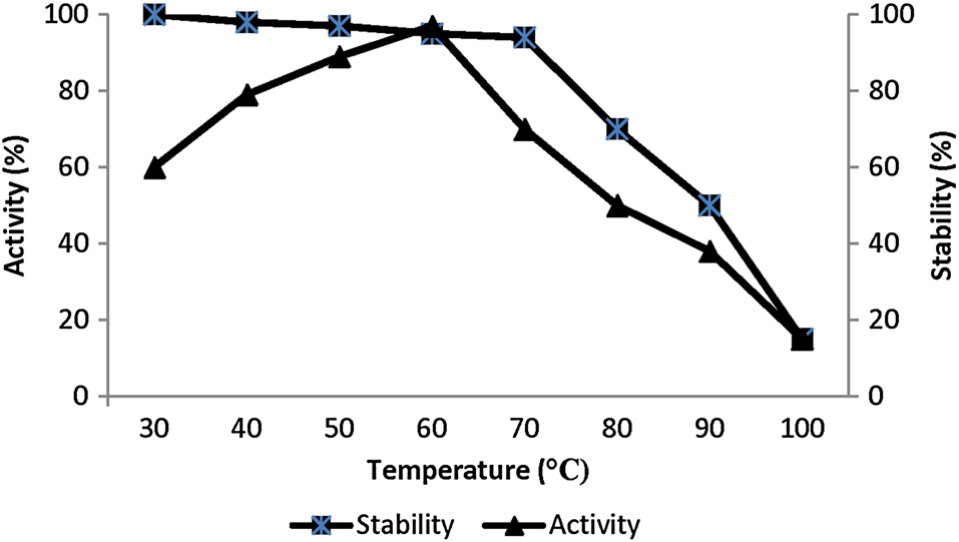
Effect of temperature on activity and stability of alkaline protease of *Bacillus* *caseinilyticus*

In the present study, enzyme activity gradually increased from temperature 30–60 °C, but, after 60 °C, it showed decrease in activity. However, the presence of 10 mM CaCl_2_ decreased it activity to some extent. Enzyme retained around 98 % residual enzyme activities at 60 °C in the presence of 10 mM CaCl_2_ (*P* ≤ 0.1) (Fig. 4).

The active site titration of the enzyme with PNPGB determined the concentration of the enzyme to be 15.0 μm (based on OD measured), with 40 % active sites.

### Effect of inhibitors and metal ions on enzyme activity

The tested alkaline protease was almost undisturbed in the presence of EDTA showing an increased relative activity of 96 %, and least activity (30 %) in the presence of Na^2+^. The results (Table 3) also showed that the enzyme was completely inhibited by serine protease inhibitor (PMSF) at both 5 and 10 mM concentrations, suggesting its serine nature (Adinarayana et al. 2003). Among the metal ions checked on the enzyme activity, Ca^2+^ and Mg^2+^ ions increased relative activity up to 82 and 85 % respectively. A serine protease from *Bacillus subtilis* DR8806 was stimulated by K^+^, Ca^2+^, Mg^2+^ and Fe^2+^ at 10 mM concentration up to 134, 129, 128 and 112 %, respectively. Whereas, Na^+^ ions had no significant effect on enzyme activity (Farhadian et al. 2015). Metal-decreasing activity in the presence of Co^2+^, Ni^2+^ was observed by Farhadian et al. (2015), Shah et al. (2010), Priya et al. (2014), Ibrahim et al. (2011) and Jain et al. (2012). In contrast, our results showed a moderate activity in presence Zn^2+^, Co^2+^ and Ni^2+^ ions. The enzyme from this study showed less activity in the presence of Fe^2+^ and Cu^2+^ ions.

**Table 3:** Effect of inhibitors, metal ions (5 mM), substrates (1 mg/ml) and organic solvents (10 %) on alkaline protease activity produced by *Bacillus* *caseinilyticus*

Inhibitor/activator	Relative activity %
PMSF	0
EDTA	96 ± 3.9
CuCl_2_	44 ± 4.1
ZnCl_2_	78 ± 2.1
MgCl_2_	85 ± 4.0
NaCl	30 ± 2.7
CaCl_2_	82 ± 3.6
FeCl_2_	54 ± 3.9
CoCl_2_	62 ± 2.1
NiCl_2_	58 ± 4.2
Casein	94 ± 2.5
Bovine serum albumin	40 ± 4.1
Gelatin	64 ± 3.7
Egg albumin	85 ± 2.9
Hexane	99.7 ± 2.6
Acetone	90.93 ± 1.8
Benzene	95.8 ± 2.2
Toulene	89.6 ± 3.5
Chloroform	69.8 ± 3.9

All the results were presented as mean ± SD

### Effect of different substrates on enzymatic activity

Various substrates were tested to study the activity of alkaline protease. Among casein, bovine serum albumin, gelatin and egg albumin, protease showed high activity (94 %) on casein (Table 3). However, the enzyme could hydrolyze several other proteins like BSA, gelatin and egg albumin, which is an important characteristic of this alkaline protease. Adinarayana et al. (2003) reported similar finding that casein was a good substrate for protease produced by *B. subtilis*. Our result was in consistent with finding of Dubey et al. (2006) which showed casein was the most preferable substrate. They had shown the activity in the presence of wheat gluten was just 20 %, in comparison with casein. Furthermore, proteases produced by *B. halodurans* 373 CAS6 (Annamalai et al. 2013), *B. cereus* TKU006 (Wang et al. 2009) and *B. subtilis* DR8806 (Farhadian et al. 2015) showed the most activity towards casein as a substrate.

### Effect of polar and non polar solvents on enzymatic activity

The effect of different organic solvents on stability revealed that the enzyme activity had no effect with hexane (99.7 %), whereas, moderate effect was found with acetone (90.9 %), benzene (95.8 %), and toluene (89.6 %); whereas, chloroform lowered (69.8 %) the alkaline protease activity (Table 3). Similarly alkaline protease produced by marine bacterium *B. firmus* CAS 7 was quite stable in the presence of anionic and non-ionic surfactants and organic solvents (Neelamegam et al. 2014). Hexane, toluene and butanol at 10 % (v/v) led to decrease the activity and the presence of DMSO in contrast increase the serine protease by *B. subtilis* DR8806. In contrast, ethanol and methanol at all tested concentrations strongly enhanced the enzyme activity as compared to the control (Farhadian et al. 2015). Jain et al. (2012) showed the protease was activated with solvents, such as, DMSO and hexane. In addition, Rai and Mukheerje demonstrated subtilisin-like serine protease isolated from *B. subtilis* DM04 increased its activity with hexane, methanol and ethanol (Rai and Mukherjee 2010). A protease from *B. pumilus* 115b has been inactivated by toluene and benzene (Rahman et al. 2007).

### Effect of different surfactants and oxidizing agent on activity of alkaline protease

A good detergent protease must be compatible and stable with all commonly used detergent compounds, such as, surfactants, bleaches, oxidizing agents and other additives which might be present in the formulation (Gupta et al. 2005). Hence different surfactants and oxidizing agent in different concentrations were tested for their effect on the alkaline protease activity. Among the tested (Tween 20, SDS and H_2_O_2_), H_2_O_2_ was showing very less effect on the activity of alkaline protease showing a relative activity of up to 91 and 86 % after treatment with 0.5 and 1 % H_2_O_2_, respectively (Table 4). In a similar report, Joo et al. (2003) reported that *Bacillus clausii* 1-52 protease exhibited relative activity of up to 114 % after treatment with 1 % H_2_O_2_. The protease produced from *Bacillus alcalophilus* TCCC11004 was exhibiting 69.2 % activity in the presence of 1 % H_2_O_2_ (Cheng et al. 2010). Our results showed that SDS might have improved the interaction of enzyme with substrate and this could result in increased relative activity than Tween 20. Cheng et al. (2010) reported that the protease produced from *Bacillus alcalophilus* TCCC11004 was stable in 0.5 % SDS and retained 70.3 % of its initial activity after 1 h of incubation.

**Table 4:** Effect of different surfactants and oxidizing agent on stability of alkaline protease produced by *Bacillus* *caseinilyticus*

Surfactants/oxidizing agents	Concentration (%)	Relative activity %
Tween 20	0.5	66 ± 3.9
	1.0	42 ± 2.1
SDS	0.5	81 ± 2.9
	1.0	55 ± 3.5
H_2_O_2_	0.5	91 ± 4.0
	1.0	86 ± 2.6

All the results were presented as mean ± SD

### Application of purified protease on blood stain removal

**Fig. 5:**
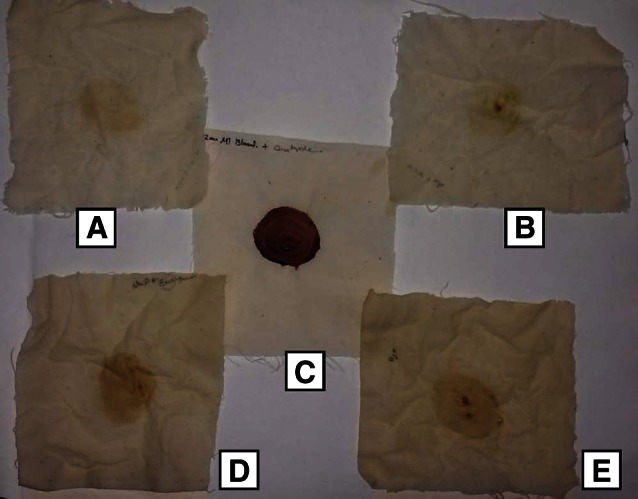
Application of purified protease of *Bacillus caseinilyticus* on blood stain removal. *A* Cloth washed with protease (2 ml) plus surf excel detergent (5 mg/ml), *B* cloth washed with surf excel detergent (5 mg/ml) alone, *C* control cloth with blood stain *D* cloth washed with protease (2 ml) alone, *E* cloth washed with sterile distilled water

In the blood stain removal experiment, blood stains were removed completely within 30 min in set A [combination of detergent (5 mg/ml) and purified enzyme (2 ml)], whereas, blood stains were not removed completely when only the enzyme was used (set D) (Fig. 5). Banerjee et al. (1999) reported that thermostable alkaline protease from *Bacillus brevis* removed blood stains with combination of 7 mg/ml detergent + enzyme (2 ml) for 25 min and similarly, in an another report, blood stains were removed completely within 15 min in combination of detergent (6 mg/ml) and purified enzyme from *Virgibacillus halodenitrificans* RSK CAS1, whereas, it took 20 min when only the enzyme was used (Ramamoorthy et al. 2014). Thus, our results clearly indicate that the addition of enzyme to a commercial detergent would enhance the performance of the detergent significantly in the process of removing blood stains and could possibly be used in the manufacture of cleaning detergent at the industrial scale.

## Conclusion

The enzyme (alkaline serine protease) produced by *Bacillus caseinilyticus* was purified using ammonium sulphate precipitation method (60 %), dialysis and finally with DEAE cellulose column chromatography. The purified protein has a molecular weight of 66 kDa. PMSF completely inhibited the alkaline protease activity indicating it as a serine protease. The purified enzyme showed its activity and stability at high temperatures and pH range. The enzyme also showed its varied stability in the presence of different inhibitors, metal ions, surfactants, oxidizing agent, polar, non polar solvents and had the capacity of hydrolyzing different substrates indicating the possibility of commercial exploitation of the alkaline serine protease in the laundry detergent industry.
